# Association of Inclusion of More Black Individuals in Lung Cancer Screening With Reduced Mortality

**DOI:** 10.1001/jamanetworkopen.2021.19629

**Published:** 2021-08-24

**Authors:** Ashley E. Prosper, Kosuke Inoue, Kathleen Brown, Alex A.T. Bui, Denise Aberle, William Hsu

**Affiliations:** 1 Department of Radiological Sciences, David Geffen School of Medicine at UCLA, Los Angeles, California; 2 Medical & Imaging Informatics Group, Department of Radiological Sciences, David Geffen School of Medicine at UCLA, Los Angeles, California; 3 Fielding School of Public Health, Department of Epidemiology, University of California, Los Angeles; 4 Department of Social Epidemiology, Kyoto University Graduate School of Medicine, Kyoto, Japan; 5 Department of Bioengineering, UCLA Samueli School of Engineering, Los Angeles, California

## Abstract

**Importance:**

The potential to achieve greater reductions in lung cancer mortality than originally estimated by the National Lung Screening Trial with the inclusion of more Black participants stresses the importance of improving access to lung cancer screening for Black current and former smokers, a population presently with the highest lung cancer morbidity and mortality.

**Objective:**

To estimate lung cancer and all-cause mortality reductions achievable with lung cancer screening via low-dose computed tomography (LDCT) of the chest in populations with greater proportions of Black screening participants than seen in the original NLST cohort.

**Design, Setting, and Participants:**

This cohort study was conducted as a secondary analysis of existing data from the National Lung Screening Trial, a large national randomized clinical trial conducted from 2002 through 2009. NLST participants were current or former smokers, aged between 55 and 74 years, with at least 30 pack-years of smoking history and less than 15 years since quitting. Cox proportional hazard models were used to estimate the hazard ratios (HRs) and 95% CIs of lung cancer mortality and all-cause mortality according to LDCT screening compared with chest radiograph screening. Using a transportability formula, we estimated outcomes for LDCT screening among hypothetical populations by varying the distributions of Black individuals, women, and current smokers. Data were analyzed between September 2020 and March 2021.

**Exposures:**

Lung screening with LDCT of the chest compared with chest radiography.

**Main Outcomes and Measures:**

Lung cancer mortality and all-cause mortality.

**Results:**

This study included a total of 53 452 participants enrolled in the NLST. Of 2376 Black individuals and 51 076 non-Black individuals, 21 922 (41.0%) were women and the mean (SD) age was 61.4 (5.0) years. Over a median (interquartile range) follow-up of 6.7 (6.2-7.0) years, LDCT screening among the synthesized population with a higher proportion of Black individuals (13.4%, mirroring US Census data) was associated with a greater relative reduction of lung cancer mortality (eg, Black individuals: HR, 0.82; 95% CI, 0.72-0.92; vs entire NLST cohort: HR, 0.84; 95% CI, 0.76–0.96). Further reductions in lung cancer mortality by LDCT screening were found among a hypothetical population with a higher proportion of men or current smokers, along with a higher proportion of Black individuals (ie, 60% Black participants; 20% to 40% women) (HR, 0.68; 95% CI, 0.48-0.97).

**Conclusions and Relevance:**

The potential to achieve greater reductions in lung cancer mortality than originally estimated by the NLST with the inclusion of more Black participants stresses the critical importance of improving access to lung cancer screening for Black current and former smokers.

## Key Points

**Question:**

Could improved access to lung screening among Black individuals achieve lung cancer mortality benefits greater than those estimated by the National Lung Screening Trial (NLST)?

**Findings:**

In this cohort study analyzing results from a randomized clinical trial, increasing the prevalence of Black individuals in hypothesized screening populations was associated with greater relative reductions of lung cancer and all-cause mortality than observed in the original NLST cohort.

**Meaning:**

These results suggest the critical importance of improving lung screening access for Black current and former smokers.

## Teaser

This cohort study of the National Lung Screening Trial uses a transportability formula to estimate whether improved access to low-dose computed tomographic screening among Black individuals would improve mortality rates over trial estimates.

## Introduction

Lung cancer is the third most common cancer in the US, and the leading cause of cancer-related death.^1^ In the US, Black individuals are disproportionately affected by cancer, experiencing the highest rate of death and lowest rates of survival for most cancers. This statistic is particularly true of lung cancer, with Black men experiencing higher rates of lung cancer death than any other racial or ethnic group.^2^

The landmark National Lung Screening Trial (NLST), a large randomized clinical trial involving 53 452 participants enrolled between 2002 and 2004, demonstrated a 20% reduction in lung cancer mortality with annual lung cancer screening using low-dose computed tomography (LDCT) of the chest when compared with chest radiograph.^3^ In light of the results of the NLST and a 2014 comparative modeling study,^4^ the US Preventive Services Task Force (USPSTF) issued a grade B recommendation that current and former smokers between ages 55 and 80 years with a 30 pack-years or more smoking history and with 15 or less years since quitting receive annual lung cancer screening with LDCT. The Center for Medicare & Medicaid Services followed this with a national coverage decision providing lung cancer screening as a covered benefit. Notably, since the reporting of the NLST results in 2011, additional trials have supported the NLST’s findings including the Nederlands–Leuvens Longkanker Screenings Onderzoek (NELSON)^5^ and Multicentric Italian Lung Detection (MILD)^6^ trials, which demonstrated 24% and 39% reductions in lung cancer mortality with LDCT screening vs no screening, respectively. Most recently, the USPSTF has expanded lung screening eligibility to include current and former smokers between ages 50 and 80 years with 20 pack-years or more of smoking history and 15 or less years since quitting.^7^

The benefits of lung screening with LDCT are now well accepted. However, to date, much of what is referenced in support of the importance of lung screening for Black current and former smokers is based on analysis of clinical screening programs.^8,9,10^ These descriptive analyses provide important insights into the effects of screening in eligible Black participants, yet clinical trials remain the criterion standard in the assessment of the efficacy of clinical interventions.

Because the NLST recognized the importance of proportionate inclusion of eligible participants from varied demographic categories, trial investigators made specific efforts early on to recruit Black participants. Seven NLST-American College of Radiology Imaging Network (ACRIN) sites were identified with strong performance in overall recruitment, successful enrollment of underrepresented minority populations, and use of a location centered in culturally diverse communities. These recruitment sites developed strategic plans for the enrollment of individuals from racial and ethnic minority groups, which required evaluating potential barriers to recruitment and collaborating on solutions. As a result, institutions with specific minority recruitment plans enrolled higher numbers of Black participants (ie, 9.5% as compared with 2.0% in institutions without specific recruitment plans).^11^ In total, among the 53 452 participants in the NLST, 2376 (4.4%) self-identified as Black.^3^

Still, the relatively low overall participation of Black individuals in the NLST has been identified as a potential barrier for extrapolating these encouraging results to populations with higher proportions of Black individuals.^12^ Subanalysis of the NLST by race revealed that these participants reported a higher prevalence of poor prognostic indicators associated with worse lung cancer outcomes than White participants, including current smoker status (although they reported lower overall cigarette consumption), being unmarried, lacking completion of a college degree, and higher numbers of comorbidities. Despite an increased prevalence of these poor prognostic indicators among Black participants, those who received lung cancer screening with LDCT experienced the greatest reduction in lung cancer mortality of any racial/ethnic group. Black participants experienced a lung cancer mortality hazards ratio (HR) of 0.61 vs 0.86 in White participants, and 0.72 in other/nonreported individuals. Black participants also experienced an all-cause mortality HR of 0.81 vs 0.95 in White participants.^13^ However, subgroup analyses of clinical trials by race limit interpretation of an intervention’s effect to specific racial/ethnic groups (ie, all Black and all White participant groups). Transportability allows for evaluation of an intervention across a population with different proportions of individuals from various racial groups. Using transportability, we can further evaluate the potential benefit of LDCT screening among populations with higher proportions of Black individuals (than the 4.4% enrolled in the NLST) by mirroring populations of interest, such as for all adults in the US. Therefore, with the use of a transportability formula, in this study we estimated the effect of LDCT screening on lung cancer and all-cause mortality reduction across populations with demographics that significantly differ from the original NLST population.

## Methods

### Data Sources and Study Population

The NLST was a multicenter randomized clinical trial conducted to assess the clinical effectiveness of lung screening with LDCT of the chest. The NLST included participants aged between 55 and 74 years at the time of randomization with a history of cigarette smoking of at least 30 pack-years and current smoker status or a quit date within the previous 15 years. A total of 53 452 participants were enrolled at 33 screening centers across the US between August 2002 to April 2004 and randomly assigned to 3 rounds of annual screening with LDCT screening or chest radiograph screening at a 1:1 ratio. More details in the NLST protocol can be found in prior literature.^14^ The NLST was approved by institutional review at each of the participating 33 sites, and participants were enrolled in the original study with written informed consent. Deidentified NLST data were obtained through an application to the National Cancer Institute’s Cancer Data Access System. The University of California, Los Angeles institutional review board determined this study to be category 4 exempt. Study data were analyzed from September 1 through February 28, 2021. Our study followed the reporting requirements of the Strengthening the Reporting of Observational Studies in Epidemiology (STROBE) reporting guideline.

### Measurements

Participants self-reported demographic characteristics at baseline, including age, sex, race and ethnic group (White, Black, or other), education status (less than college, college or higher, other), marital status (single, married, widowed or divorced), smoking status (current or former smokers), and pack-years of smoking. Mortality data were obtained through annual questionnaires and searches on the National Death Index. Participants were followed from the time of entry into the study until death, loss to follow-up, or through the end of the study on December 31, 2009 (the original NLST publication set the final status of lung cancer mortality at January 15, 2009).

### Statistical Analyses

Cox proportional hazard models were used to estimate the hazard ratio of lung cancer mortality and all-cause mortality according to LDCT (vs chest radiograph) screening. To assess changes in lung cancer mortality and all-cause mortality derived from the NLST population associated with application to the hypothesized target populations, we applied a transportability formula. Transportability is a statistical approach that allows for the extrapolation of results from a randomized clinical trial to a target population in which an intervention is being considered using a combination of results from the original trial participants and data on the background characteristics of the target population.^15^

In this formula (the inverse-odds weighting approach), we emulated the target population from the original NLST participants using the weights created by the odds of being in the NLST as opposed to the target population. Additional details on the application of transportability can be found in eMethods in the Supplement or elsewhere.^15,16^

Aiming to demonstrate the effects of race, sex, and smoking status on mortality reduction with LDCT screening across a synthetic population, we applied a transportability formula to NLST data in 3 settings. First, we transported the estimated effect from the NLST population to the hypothesized target populations by varying the distribution of Black individuals. Second, we transported the estimated effect to the hypothesized target populations by varying the distributions of Black individuals and sex simultaneously. And third, we transported the estimated effect to the hypothesized target populations by varying the distributions of Black individuals and smoking status simultaneously. The 95% CIs were calculated by repeating the analyses on 200 bootstrapped samples. All statistical analyses were performed with R version 4.0.2 (R Project for Statistical Computing).^17^

## Results

Of 53 452 participants enrolled in the NLST, 21 922 (41.0%) were women and the mean (SD) age was 61.4 (5.0) years; median (interquartile range) follow-up was 6.7 (6.2-7.0) years. Compared with non-Black participants, at trial enrollment Black participants were more likely to be women (1076 [45.3%] vs 20 846 [40.8%]), less educated (less than college: 1250 [52.6%] vs 22 145 [43.4%]), single or widowed/divorced (widowed or divorced: 1181 [49.7%] vs 13 885 [27.2%]), and current smokers (1578 [66.4%] vs 24 182 [47.3%]), and have fewer pack-years of smoking (mean [SD] years: 48.9 [19.0] vs 56.3 [24.1]) (Table).

**Table.** Demographic Characteristics of the Study Population in the National Lung Screening Trial (NLST)

Characteristics	Participants, No (%)	
	Black (n = 2376)	Non-Black (n = 51 076)
Age, mean (SD), y	60.5 (4.8)	61.5 (5.0)
Sex		
Men	1300 (54.7)	30 230 (59.2)
Women	1076 (45.3)	20 846 (40.8)
Ethnic group		
Hispanic	15 (0.6)	920 (1.8)
Non-Hispanic	2341 (98.5)	49 777 (97.5)
Others or missing^a^	20 (0.9)	379 (0.7)
Education status		
Less than college	1250 (52.6)	22 145 (43.4)
College or higher	1070 (45.0)	27 753 (54.3)
Others or missing	56 (2.4)	1178 (2.3)
Marital status		
Single	265 (11.2)	2193 (4.3)
Married	910 (38.3)	34 679 (67.9)
Widowed or divorced	1181 (49.7)	13 885 (27.2)
Missing	20 (0.8)	319 (0.6)
Smoking status		
Former	798 (33.6)	26 894 (52.7)
Current	1578 (66.4)	24 182 (47.3)
Pack-years of smoking, mean (SD), y	48.9 (19.0)	56.3 (24.1)

^a^ Includes participants who answered unknown, did not complete the form, or refused to answer.

**Figure 1.**
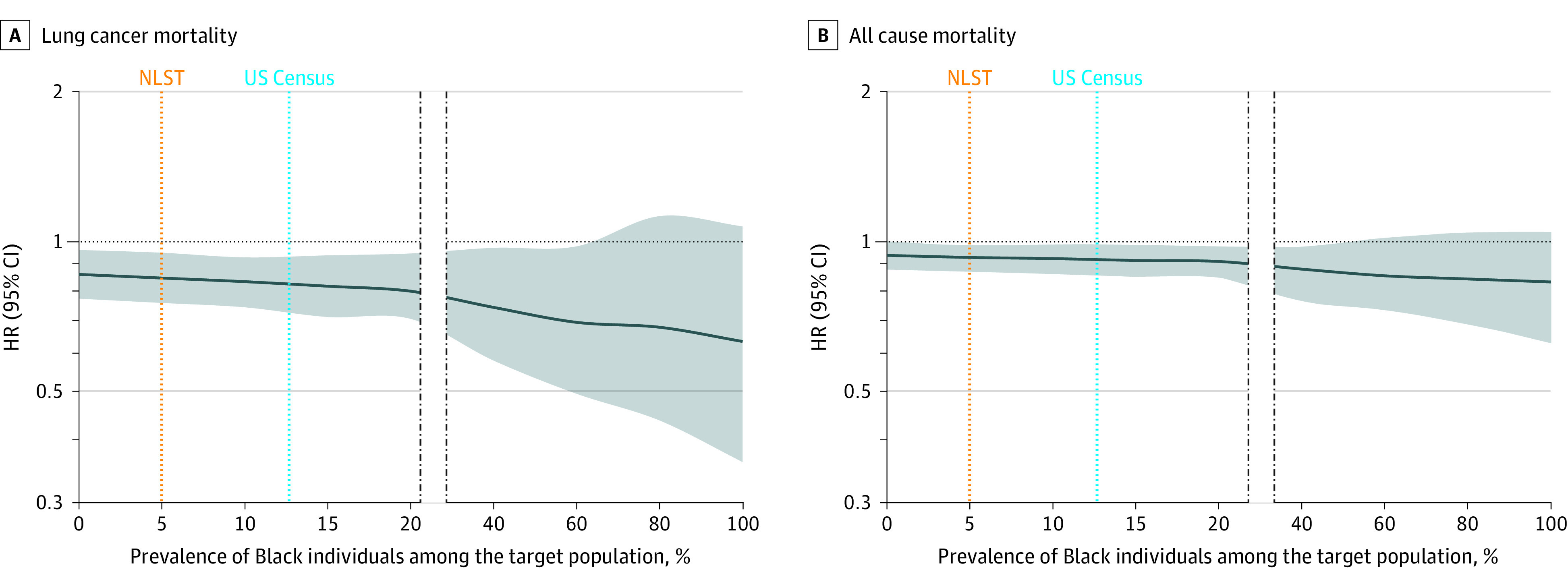
Association Between Lung Screening With LDCT and Mortality Rates When Varying Hypothetical Distributions of the Black Population HR indicates hazard ratios; LDCT, low-dose computed tomography; NLST, National Lung Screening Trial. The shaded area represents 95% CIs for mortality risk. Estimated mortality reductions are 16% in NLST (orange line) and 18% for the US Census (blue line) in panel A, and 6.7% in NLST and 8% for the census in panel B.

Increasing the prevalence of Black individuals in our hypothesized external populations resulted in an increased relative reduction of lung cancer mortality with LDCT screening (Figure 1) when compared with the 16% reduction in lung cancer mortality seen in the NLST (using the extended analysis period of December 31, 2009). For example, in a population comprising 13.4% Black individuals (ie, mirroring US Census data^18^), the relative reduction in lung cancer mortality across the population was 18% (95% CI, 8%-28%), an increase from 16% (95% CI, 4%-24%) in the NLST cohort that included 2376 (4.4%) Black individuals. Among a population comprising 46.3% Black individuals (mirroring demographic data from Washington, District of Columbia^18^), we found a further reduction in lung cancer mortality by LDCT screening (26%; 95% CI, 5%-42%). Similarly, although more subtly, increasing the prevalence of Black individuals in our hypothesized external populations to 13.4% and 46.3% resulted in an increased relative reduction of all-cause mortality with LDCT screening to 8% (95% CI, 1%-15%) and 13% (95% CI, 0%-25%), respectively.

**Figure 2.**
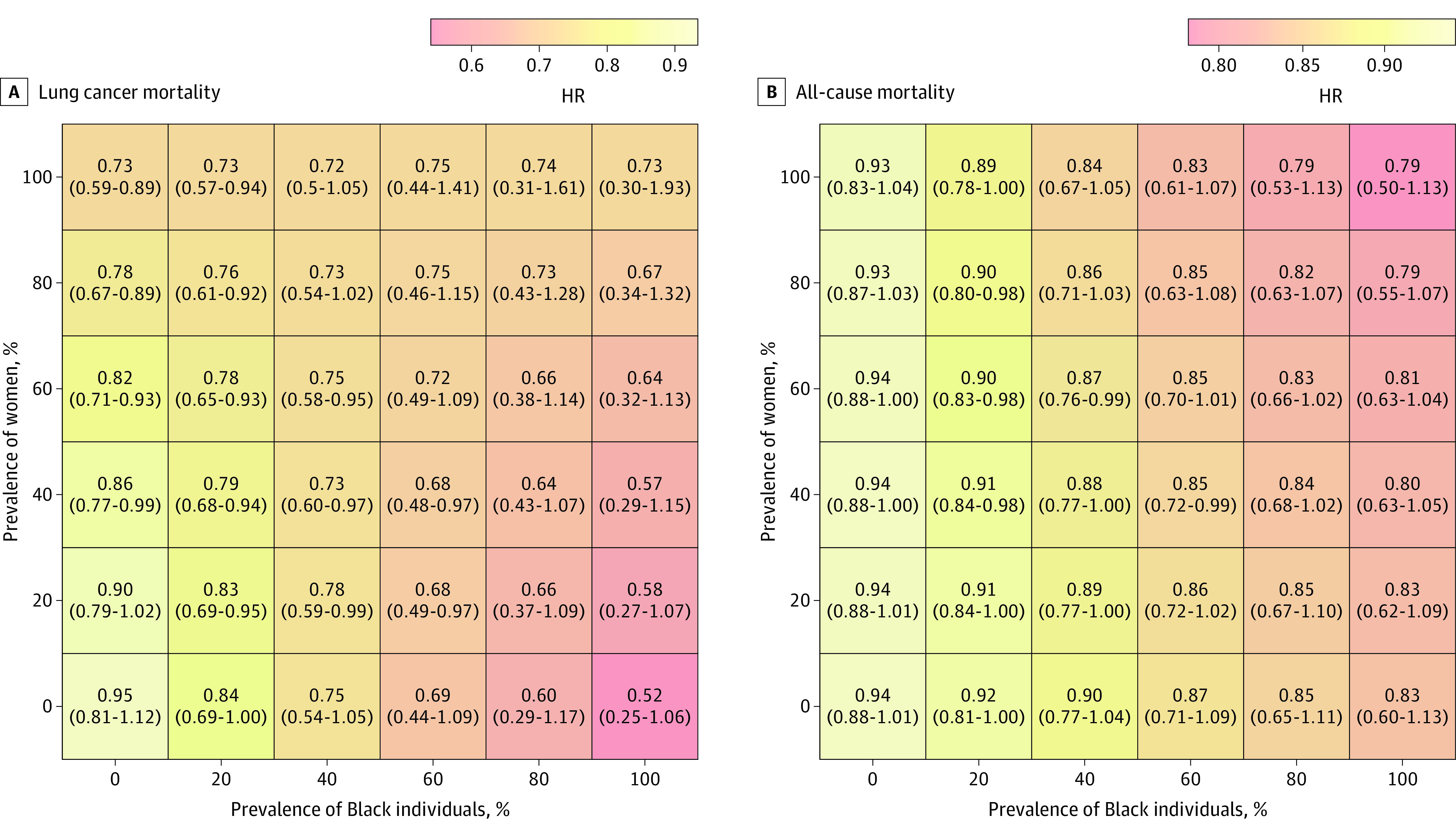
Heat Map of Association Between Lung Screening With LDCT and Mortality Rates When Varying Hypothetical Distributions of the Black Population and Sex HR indicates hazard ratio; LDCT, low-dose computed tomography.

Varying the distribution of race and sex simultaneously, we also saw increased reductions in lung cancer mortality with greater proportions of Black individuals and lower proportions of female participants (Figure 2). For instance, the greatest statistically significant lung cancer mortality benefit across the population (HR, 0.68; 95% CI, 0.48-0.97) was seen with a population that was 60% Black and between 20% and 40% female. Notably, reduction in all-cause mortality increased with higher proportions of Black individuals, regardless of the proportion of female participants.

**Figure 3.**
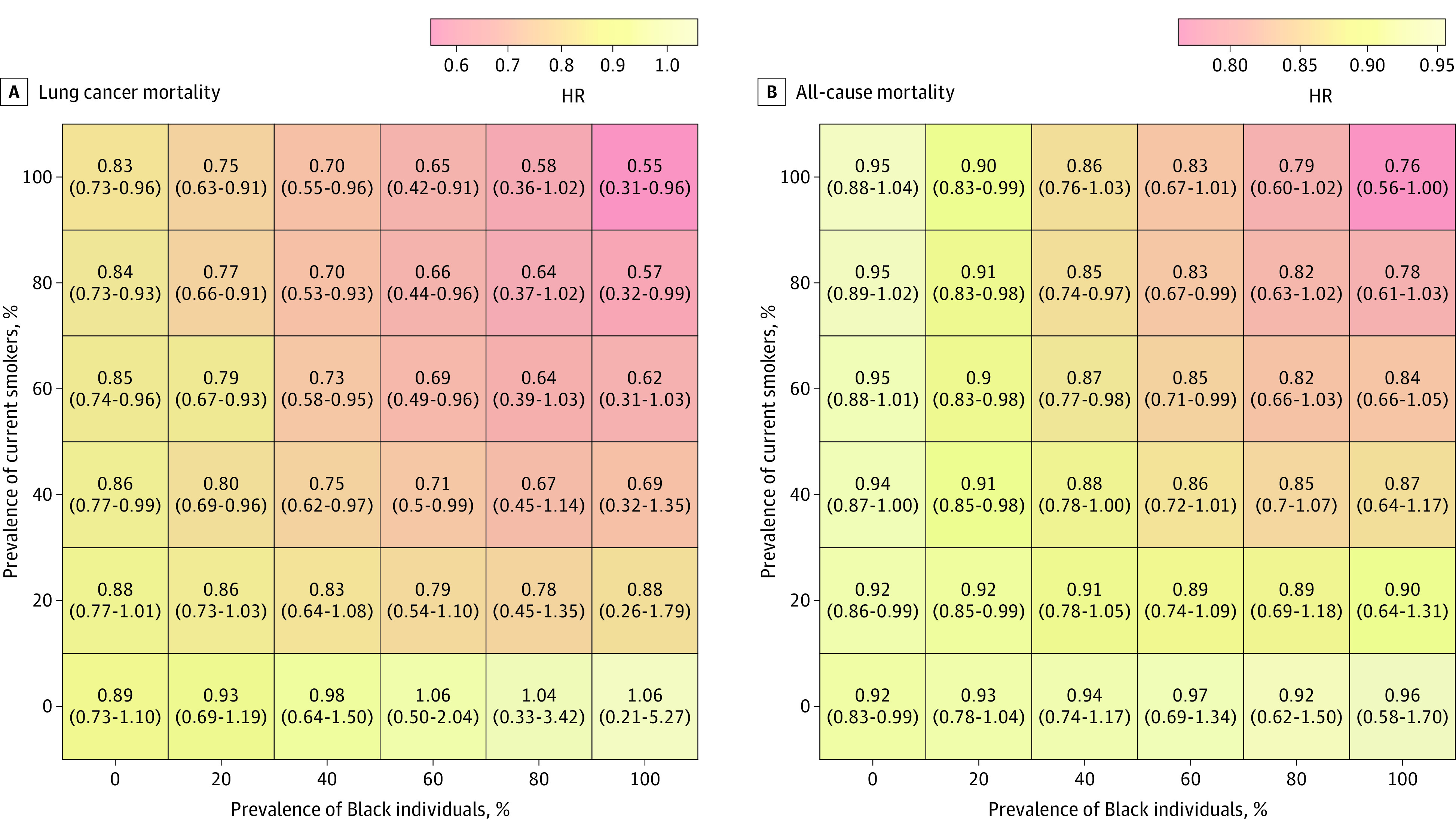
Heat Map of Association Between Lung Screening With LDCT and Mortality Rates When Varying Hypothetical Distributions of the Black Population and Smoking Status HR indicates hazard ratio; LDCT, low-dose computed tomography.

By varying the distribution of race and smoking status simultaneously, we observed the greatest reduction in lung cancer mortality by increasing the number of Black individuals and current smokers, with up to a 45% reduction in lung cancer mortality (adjusted HR, 0.55; 95% CI, 0.31-0.96) in a population with 100% Black current smokers (Figure 3). Among all of the synthetic populations with the same sample size as the original NLST, the greatest reduction in all-cause mortality was seen with a population comprising 100% Black participants and current smokers, but the result was not statistically significant (adjusted HR, 0.79; 95% CI, 0.50-1.13).

## Discussion

Extrapolation of NLST results to synthetic populations with higher proportions of Black individuals using a transportability formula suggests that the lung cancer mortality reduction achievable with LDCT screening is potentially greater than originally reported. Creating a synthetic population that mirrors the proportion of Black individuals in the US (without changing the distribution of other variables from the original NLST population), we would expect a lung cancer mortality reduction of 18%.^18^

It is important to note that the mortality reduction we have modeled via transportability is limited by the original trial data to which it is applied. A review of the transported effect of LDCT screening with varied proportions of Black participants (Figure 1) highlights these limitations. Given that only 4.4% of the original NLST population were Black participants, the confidence interval in our predicted estimates crosses 1.0, and data becomes insufficient for further extrapolation at a proportion of 60% Black participants. Equally important to understand is that the maximal lung cancer and all-cause mortality reduction that can be theoretically achieved with our synthetic populations (at a proportion of 100% Black participants) is almost identical to the mortality reduction achieved in the NLST cohort of 2376 Black participants, or 39%.

Transportability analysis is a powerful tool that helps us to posit clinical trial results in synthetic populations that better mirror real-world patient populations. In these examples, transportability allowed us to estimate the effect of LDCT screening on lung cancer and all-cause mortality reduction across several hypothetical populations with varied proportions (ie, from 0% to 100%) of Black individuals, women, and current smokers. In contrast, subgroup analysis, the most common approach used when focusing on a specific population, would have only allowed us to estimate the effect among the population with 0% or 100% proportions of a specific variable (eg, among Black individuals, women, current smokers), indicating that transportability approach may be able to provide more detailed and flexible information than attainable through subgroup analysis. Moreover, while the present study only varies the prevalence of 1 variable (ie, sex or smoking status) in addition to that of Black individuals for simple illustration, the transportability approach allows us to include as many measured variables as necessary, and to quantify the intervention effect across any target populations of interest under the required causal assumptions.^15,16^ The transportability approach may also be applied in other important topics, such as the cost-effectiveness of LDCT screening. Given the heterogeneous incremental cost-effectiveness ratios of LDCT screening across individuals’ demographic characteristics,^19,20^ future studies are needed to extend the findings of its cost-effectiveness analysis to the target population of interest using transportability analysis.

### Limitations

This study had several limitations. Notwithstanding the advantages of transportability, it must be understood that this statistical method is not a replacement for equitable and inclusive recruitment of diverse groups of clinical trial participants. Had the NLST research team not made a concerted effort to increase enrollment of Black participants through a partnership with 7 NLST-ACRIN sites, our ability to apply transportability to original NLST data would likely have been much more statistically limited. Thus, the transportability analysis does not completely negate the need for future clinical trials, but is a statistical tool that provides valuable information for future trials concerning (1) what kind of populations would get the benefit from the intervention and (2) the estimated extent of expected intervention effects.

We additionally recognize that accurately reflecting the racial demographics of our population with clinical trial recruitment is only one of the limitations in extrapolating clinical trial results to real-world settings. Clinical trial participants receive careful surveillance and, as a result, have higher levels of adherence than that seen in clinical practice. NLST participants achieved a greater than 90% adherence rate to screening,^3^ much higher than those reported in clinical programs.^21,22^ Despite evidence of mortality benefit with lung screening and the fact that screening is a covered benefit in eligible individuals by both private insurers and Medicare, the utilization of, and adherence to, lung cancer screening remains suboptimal, and far below the adherence seen in the NLST. A review of the American College of Radiology Lung Cancer Screening Registry in 2016 revealed a woefully low 1.9% utilization rate among 7.6 million eligible smokers.^23^ Moreover, while lung screening is currently underutilized by eligible participants as a whole, Black current and former smokers are disproportionately challenged in entry and adherence to lung cancer screening, are less likely to receive lung cancer screening, and experience longer times to follow-up than White patients.^12,24^ Cited barriers to cancer screening for eligible individuals include limited access, fear, fatalism, mistrust of the medical system, and experiences with racism.^8,25,26^ In addition, evidence revealed the original USPSTF lung screening eligibility criteria to be insufficiently inclusive of Black current and former smokers, who develop lung cancer at younger ages with a lower cumulative pack-year smoking history than current eligibility cutoffs.^10^ The newly revised LDCT screening eligibility guidelines from the USPSTF mitigate the exclusion of Black smokers from potential screening benefits by reducing the eligibility age to 50 years and smoking intensity to 20 or more pack-years.^7^ As we continued to improve risk-based criteria for screening, this approach increases the number of Black individuals at highest risk of lung cancer.

## Conclusions

Even with the myriad barriers to enrollment in and ultimately adherence to lung screening with LDCT, extrapolation of NLST results to diverse populations with increased proportions of Black screening participants is nonetheless encouraging. The potential to achieve greater reductions in lung cancer mortality than originally estimated by the NLST with the inclusion of more Black participants stresses the critical importance of working to improve access to lung cancer screening for Black current and former smokers.

## Notes

Supplement. **eMethods.** Transportability Analysis (the Inverse-odds Weighting Approach) to Estimate the Intervention Effect of LDCT Screening to Reduce Mortality Across Hypothetical Target Populations
