# Supplementary material for: Association of Inclusion of More Black Individuals in Lung Cancer Screening With Reduced Mortality
Source: JAMA Netw Open. 2021 Aug 24;4(8):e2119629. doi: 10.1001/jamanetworkopen.2021.19629 (PMC8385597; doi:10.1001/jamanetworkopen.2021.19629)
Supplement: Supplement. — eMethods. Transportability Analysis (the Inverse-odds Weighting Approach) to Estimate the Intervention Effect of LDCT Screening to Reduce Mortality Across Hypothetical Target Populations [file jamanetwopen-e2119629-s001.pdf]

Supplemental Online Content

Prosper AE, Inoue K, Brown K, Bui AAT, Aberle D, Hsu W. Association of inclusion of more Black individuals in lung cancer screening with reduced mortality. *JAMA Netw Open.* 2021;4(8):e2119629. doi:10.1001/jamanetworkopen.2021.19629

eMethods. Transportability Analysis (the Inverse-odds Weighting Approach) to Estimate the Intervention Effect of LDCT Screening to Reduce Mortality Across Hypothetical Target Populations

This supplemental material has been provided by the authors to give readers additional information about their work.

© 2021 Prosper AE et al. *JAMA Network Open.*

eMethods. Transportability analysis (the inverse-odds weighting approach) to estimate the intervention effect of LDCT screening to reduce mortality across hypothetical target populations.

In general, transportability is a concept to apply results to a target population that partially includes or does not at all include the study population (e.g. the population of NLST-eligible non-participants). By calculating the intervention effect by LDCT screening across target populations of interest beyond the NLST trial participants, we can provide valuable information for future trials and clinical guidelines about what kind of populations would receive the most benefit if the LDCT screening is implemented.

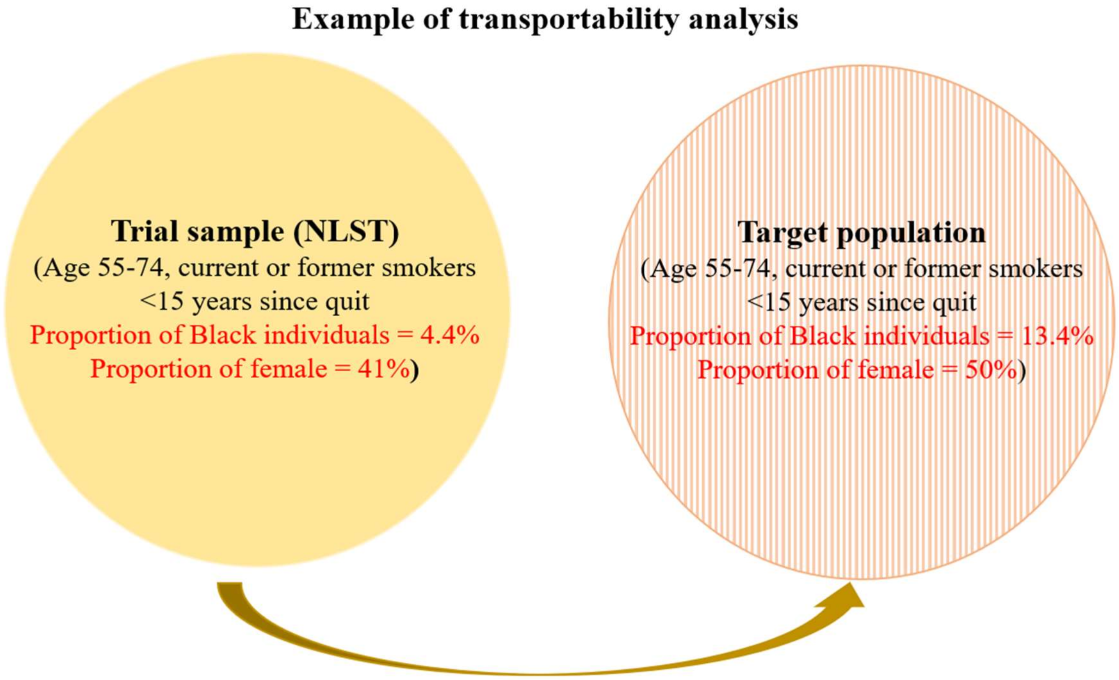

More technically, this approach extends the original NLST results to the target population of interest by comparing the distribution of baseline characteristics that modify the effect of the intervention between the original study sample and target population. In the inverse-odds weighting approach, we have the following five steps to estimate the benefit of LDCT screening to reduce mortality across hypothetical target populations with different distributions of Black individuals and female or current smokers that were derived from the original NLST trial. The

distribution of other covariates among those hypothetical target populations are assumed to remain the same as in the original trial. More details in the transportability formula can be found elsewhere.[1,2]

Step 1. Create a hypothetical target population with a different distribution of covariates (Black race and sex or smoking status) derived from the original NLST data (N=53,452).

Step 2. Using the original NLST data and the created hypothetical target population data, calculate the inverse odds of sampling weights as follows:

$$\left[\frac{Probabilit\quad of\ being\ in\ the\ target\ population\ given\ covariates}{Probability\ of\ being\ in\ the\ trial\ sample\ given\ covariates}\right] \times \left[\frac{Probability\ of\ being\ in\ the\ trial\ sample}{Probability\ of\ being\ in\ the\ target\ population}\right]$$

Step 3. Assign the weights to individuals in the original NLST data.

Step 4. Employ Cox proportional hazard models along with the assigned weights (to emulate the hypothetical target population from the original NLST participants) to estimate the benefit of LDCT screening to reduce lung cancer mortality among the hypothetical target populations.

Step 5. The 95% confidence intervals are estimated from the variability of the statistic derived from the bootstrap samples.

References:

1. Bareinboim E, Pearl J. A General Algorithm for Deciding Transportability of Experimental Results. *Journal of Causal Inference*. 2013;1(1). doi:10.1515/jci-2012-0004
2. Westreich D, Edwards JK, Lesko CR, Stuart E, Cole SR. Transportability of Trial Results Using Inverse Odds of Sampling Weights. *Am J Epidemiol*. 2017;186(8):1010-1014. doi:10.1093/aje/kwx164
